# Vasostatin Inhibits VEGF-Induced Endothelial Cell Proliferation, Tube Formation and Induces Cell Apoptosis under Oxygen Deprivation

**DOI:** 10.3390/ijms15046019

**Published:** 2014-04-09

**Authors:** Qun Shu, Wenjiao Li, Haichuan Li, Gang Sun

**Affiliations:** 1 Shanghai Changning Maternity and Infant Health Hospital, 773 Wuyi Road, Shanghai 200051, China; E-Mails: liwenjiao2008@163.com (W.L.); lihaichuan_happy@163.com (H.L.); 2 School of Life Sciences, Fudan University, 220 Handan Road, Shanghai 200433, China; E-Mail: sungang122012@163.com

**Keywords:** vasostatin, human umbilical vein endothelial cells (HUVEC), angiogenesis, mitochondrial apoptosis

## Abstract

Anti-angiogenesis treatment has been a promising new form of cancer therapy. Endothelial cells are critical for vascular homeostasis and play important roles in angiogenesis, vascular and tissue remodeling. Vasostatin, the 180 amino acid *N*-terminal fragment of the calreticulin protein, is reported to be a potent endogenous inhibitor of angiogenesis, suppressing tumor growth. However, the mechanism of these effects has not been sufficiently investigated. This study was performed to investigate the possible mechanism of vasostatin effects on primary cultured human umbilical vein endothelial cells (HUVEC). We found that vasostatin could inhibit the cell viability of HUVEC and induce cell apoptosis through mitochondrial pathways via activation of caspase-3 under oxygen deprivation conditions. Meanwhile, vasostatin also inhibited vascular endothelial growth factor-induced proliferation and tube formation of HUVEC. The possible mechanism of vasostatin-inhibited proliferation of HUVEC could be through down-regulation of endothelial nitric oxide synthase. These findings suggest that vasostatin could regulate endothelial cell function and might be used in anti-angiogenesis treatment.

## 1. Introduction

Angiogenesis plays a critical role in the growth and spread of cancer as an adequate blood supply is necessary for tumor growth and invasion in normal tissues [1,2]. Therefore, agents that block the growth of a tumor’s blood vessels promote the regression or dormancy of established tumors and anti-angiogenesis treatment has been a promising new form of cancer therapy [3].

It is known that endothelial cells are critical for vascular homeostasis and play important roles in angiogenesis, vascular and tissue remodeling. Since Pike *et al.* first reported in 1998 [4], vasostatin, the 180 amino acid *N*-terminal fragment of the calreticulin protein, has been recognized as a potent endogenous inhibitor of angiogenesis to suppress tumor growth. A growing body of evidence supports the beneficial effect of anti-angiogenesis of vasostatin. Recombinant vasostatin prevented or apparently reduced the growth of human Burkitt lymphoma, colon carcinoma, and ovarian carcinoma in murine modes [5,6]. In addition, cancer gene therapy based on the intramuscular delivery of plasmid DNA encoding vasostatin is effective in the inhibition of systemic angiogenesis and tumor growth in murine models [7]. Angiogenesis is the growth of blood vessels from the existing vasculature, and is a key factor in tumor growth and spread [8]. The protagonists of this process are endothelial cells (ECs), which are regulated by microenvironmental factors, such as hypoxia and chronic growth factor stimulation resulting in endothelial dysfunction. These factors regulate ECs proliferation, migration, organization in tubular structures, and, finally, these abnormalities in the tumor endothelium contribute to tumor growth and metastasis [9].

However, the mechanism of anti-angiogenic effects of vasostatin on endothelial cells has not been investigated sufficiently. The aim of the present study is to investigate possible mechanism of vasostatin effect on primary cultured human umbilical vein endothelial cells (HUVEC).

## 2. Results and Discussion

### 2.1. Vasostatin Inhibited Cell Viability Dose-Dependently and Induced Apoptosis of HUVEC under Oxygen-Deprivation

**Figure 1.**
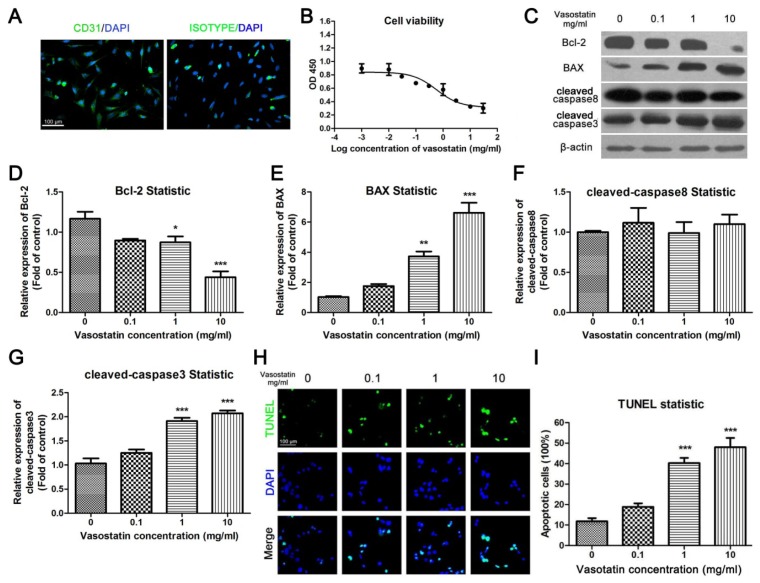
Vasostatin inhibited cell viability and induced apoptosis of HUVEC under oxygen-deprivation. (**A**) Identification of HUVEC. Primary cultured HUVEC were stained by CD31 antibody or isotype IgG (Green), the nuclei of the cells were stained blue. Scale bar = 100 μm and referred to the two panels; (**B**) Cell viability assay. Cultured HUVEC were incubated with different concentrations of vasostatin under oxygen deprivation for 24 h. The absorbance at a wavelength 450 nm was measured as relative cell viability, *n* = 6, non-linear regression was performed to calculate *IC*_50_; (**C**) Determination of apoptotic markers. The expression of Bcl-2, BAX, cleaved caspase8 and cleaved caspase3 were determined by western blot analysis. β-actin was used as a housekeeping protein; (**D**–**G**) Statistical analysis of blots. All blots were performed for at least three times. * *p* < 0.05, ** *p* < 0.01, *** *p* < 0.001 compared to control group; (**H**) TUNEL staining. TUNEL-positive cells were marked by green fluorescence. The nuclei of cells were stained by DAPI. The merged figures were shown in below. Scale bar = 100 μm and refers to all panels; (**I**) Statistical analysis of TUNEL. The apoptotic ratio of each group was calculated as the number of TUNEL-positive cells divided by total cell number (DAPI positive); *** *p* < 0.001 compared to vehicle-treated group, *n* = 10.

Primary cultured HUVEC were used in this study. Before the experiments, the purity of primary cultured HUVEC was identified by flow cytometry (CD31^+^, CD34^+^, CD45^−^, CD56^−^, data was not shown). As the marker of endothelial cells, CD31 is specifically expressed in primary cultured HUVEC [10]. Immunocytochemistry was performed to show the CD31-positive cells in total primary cultured cells. At passage 2–4, over 95% of cells were stained positively for CD31 (Figure 1A); using an isotype control IgG, less than 2% cells were positive (Figure 1A).

Cultured HUVEC were incubated with different concentrations of vasostatin under oxygen deprivation for 24 h. Then the MTT assay was used to investigate the effect of vasostatin on the viability of HUVEC. We found that vasostatin could inhibit cell viability dose-dependently (Figure 1B, *IC*_50_ = 0.57 mg/mL). To further investigate the apoptotic ratio of vasostatin-treated cells, TUNEL staining was performed. It is showed that vasostatin could also induce cell apoptosis under hypoxia conditions in a dose-dependent manner (Figure 1H,I). Meanwhile, as shown in Figure 1C, vasostatin could reduce the expression of Bcl-2, an anti-apoptosis protein, and demonstrated this significantly at a 10 mg/mL dose (Figure 1C,D). Both BAX (Figure 1C,E) and cleaved caspase3 (Figure 1C,G) were up-regulated by vasostatin dose-dependently, which suggested that vasostatin might induce HUVEC apoptosis through a BAX-caspase3 pathway. However, the expression level of cleaved caspase8 has no obvious difference between vasostatin- and vehicle-treated cells (Figure 1C,F). Therefore, the caspase8-associated mitochondrial apoptosis could not be involved in vasostatin-induced HUVEC death.

### 2.2. Vasostatin Inhibited VEGF-Induced Proliferation and Tube Formation of HUVEC

**Figure 2.**
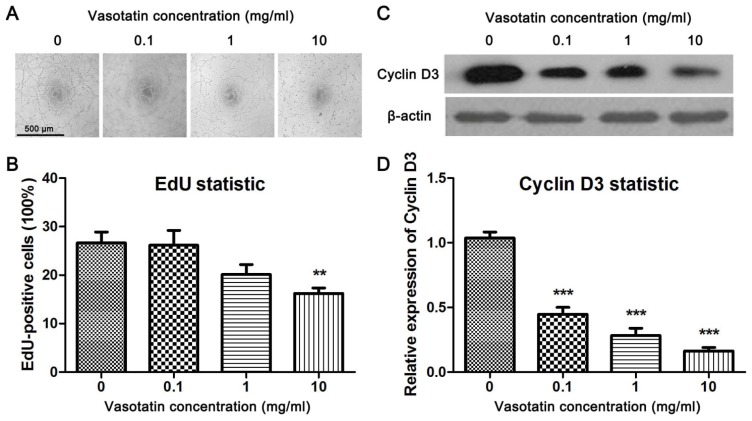
Vasostatin inhibited VEGF-induced proliferation and tube formation of HUVEC. (**A**) Tube formation. After HUVEC were treated with VEGF and different dose of vasostatin, the tube formation test was performed. Scale bar = 500 μm and referred to all panels; (**B**) EdU assay. After treatment, EdU assay was performed. The proliferation rate in each group was calculated as EdU-positive cells divided into total number of cells. ** *p* < 0.01 compared to vehicle-treated group, *n* = 10; (**C**) The expression level of cyclin D3. After treatment, the cell lysate were collected and western blot analysis was performed. β-actin was used as housekeeping protein. The blots were repeated three times. (**D**) Statistical analysis of cyclin D3 expression. The expression level of cyclin D3 in each group was analyzed according the optical density. *** *p* < 0.001, *n* = 3.

To investigate the effect of vasostatin on angiogenesis *in vitro*, the tube formation test of HUVEC was performed. We found that vasostatin could inhibit VEGF-induced tube formation of HUVEC significantly in a dose-dependent manner (Figure 2A). The proliferation of endothelial cells is associated with cell migration [11]. Therefore, the effect of vasostatin on VEGF-induced proliferation of HUVEC was studied. EdU (5-ethynyl-2′-deoxyuridine) assay was used in this experiment. As one typical proliferation assay, BrdU staining, the proliferating nuclei of cells could be marked with fluorescence probes in the same way in the EdU assay [12]. The proliferation rate of HUVEC was determined by the EdU assay and in accordance with the result of MTT, it was found that vasostatin could suppress VEGF-induced proliferation of primary cultured HUVEC at 10 mg/mL significantly (Figure 2B). Moreover, the expression of cyclin D3, one of the cell proliferation markers, was determined by western blot. It was found that VEGF-induced increasing of cyclin D3 was obviously inhibited by vasostatin (Figure 2C,D).

### 2.3. Overexpression of eNOS Reversed the Anti-Proliferation Effect of Vasostatin

**Figure 3.**
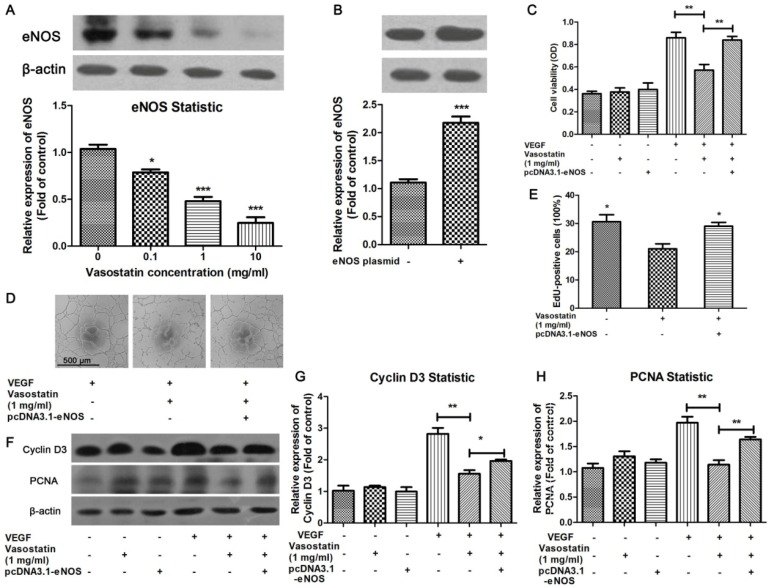
Overexpression of eNOS reversed the anti-proliferation effect of vasostatin. (**A**) Vasostatin inhibits eNOS expression at a dose of 0.1 to 10 mg/mL, the blot was repeated for three times for statistical analysis. * *p* < 0.05, *** *p* < 0.001 compared to control group; (**B**) Overexpression of eNOS on eNOS expression in HUVEC. There is about 2 fold expressed level of eNOS in transfected cells. *** *p* < 0.001 compared to negative control group; (**C**) Measurement of cell viability. After HUVEC were treated with/without 10 ng/mL of VEGF in the presence/absence of vasostatin for 24 h, the cell viability was measured by MTT assay. Some groups were preconditioned with pcDNA3.1-eNOS plasmid as indicated. ** *p* < 0.01 as indicated, *n* = 4; (**D**) Tube formation test. It was showed that overexpressed eNOS in cultured HUVEC could reverse vasostatin-induced inhibition of tube formation. Scale bar = 500 μm and referred to all panels; (**E**) EdU assay. After treatment, EdU assay was performed to investigate the effect of vasostatin on VEGF-stimulated cell proliferation. The proliferation rate in each group was calculated as EdU-positive cells divided into total number of cells. * *p* < 0.05 compared to vasostatin-treated negative plasmid-transduced group, *n* = 10; (**F**) Western blot analysis. After treatment, the expression level of cyclin D3 and PCNA was measured by western blot analysis. β-actin was used as housekeeping protein; (**G**,**H**) All blots were repeated three times for statistical analysis. * *p* < 0.05, ** *p* < 0.01 as indicated.

The possible mechanism of vasostatin-induced inhibitory effect on VEGF-induced cell proliferation and tube formation in HUVEC was investigated in this study. It has been reported that eNOS mediates the cell proliferation and migration under VEGF-stimulation [13,14]. And calreticulin has been identified to play a role in eNOS pathway in this process [15]. Therefore, to evaluate the effect of eNOS on vasostatin-induced anti-proliferation in HUVEC, we detected the eNOS expression level in the stimulation of vasostatin. It was found that vasostatin dose-dependently decreased eNOS expression (Figure 3A). Therefore, eNOS overexpression plasmid was used to up-regulate the expression level of eNOS (Figure 3B). It was found that vasostatin could significantly suppress VEGF-induced increases in cell viability of normal HUVEC, but in eNOS-overexpressed cells, this inhibitory effect was suppressed (Figure 3C). To further investigate whether this change in cell viability was through cell proliferation, EdU assay was performed. The results showed that VEGF-induced cell proliferation was inhibited by 1 mg/mL vasostatin, but overexpression of eNOS obviously abolished this effect (Figure 3E). Western blot showed that both PCNA and cyclin D3, cell proliferation markers, were activated by VEGF, and vasostatin reduced the expression of PCNA and cyclin D3 (Figure 3F–H). As expected, eNOS overexpression in HUVEC increased vasostatin-induced decreasing of PCNA and cyclin D3 expression (Figure 3F–H). Moreover, vasostatin-induced inhibition of tube formation was also reversed by eNOS overexpression (Figure 3D), suggesting that the anti-proliferation effect of vasostatin could be mainly through eNOS pathways in HUVEC.

### 2.4. Discussion

In the present study, HUVEC, a widely used experimental model for endothelial cells, were used to evaluate the anti-angiogenic mechanism of vasostatin. Firstly, the primary cultured HUVEC were identified by CD31, a marker of endothelial cells, CD31 specifically expresses in primary cultured HUVEC [16], and the cell viability was evaluated by MTT assay to investigate the effect of vasostatin. This study was performed to investigate the possible effect mechanism of vasostatin on HUVEC.

Vasostatin was purified from supernatant of an Epstein-Barr virus–immortalized cell line and identified as fragments of calreticulin. The calreticulin-derived vasostatin is structurally unrelated to other known peptide inhibitors of blood vessel integrity and angiogenesis [4], such as plasminogen-derived angiostatin, collagen-derived endostatin, and chromograin A-derived angiogenesis inhibitor [6,17]. Compared with other inhibitors of angiogenesis, vasostatin is a small, soluble, and stable molecule that is easy to produce and deliver [4]. As an angiogenesis inhibitor that specifically targets proliferating endothelial cells, vasostatin has a unique potential for cancer treatment [5,18,19]. A wide range of inhibitory activities has since been assigned to the vasostatin [20]. It was found that there was no obvious effect of vasostatin on cell viability of HUVEC after 24 h treatment under normal culture conditions (data not shown), which suggested that vasostatin has less cytotoxic effects on normal cells. Therefore, to further investigate the effect mechanism of vasostatin on angiogenesis, in the present study, we found that vasostatin could inhibit cell viability dose-dependently under oxygen deprivation conditions. It is showed that vasostatin could also induce cell apoptosis under hypoxia conditions in a dose-dependent manner. Meanwhile, at high doses vasostatin could significantly reduce the expression of Bcl-2, an anti-apoptosis protein. The expression level of BAX and cleaved caspase3 were both up-regulated by vasostatin dose-dependently. However, the expression of cleaved caspase8 has displayed no obvious change between vasostatin- and vehicle-treated cells. Therefore, the caspase8 associated mitochondrial apoptosis could not be involved in vasostatin-induced HUVEC death. It is known that BAX and cleaved caspase3 are death-promoting factors, whereas Bcl-2 protein is a death antagonist [21]. A decrease of Bcl-2 to Bax ratio is sufficient to promote apoptosis in mammalian cells and induce cell death by directly activating the mitochondrial apoptotic pathway in maturation of caspase-9*,* which in turn activates caspase3 [22,23]. Thus, these results indicated that vasostatin might induce HUVEC apoptosis through a BAX-caspase3 pathway.

On the other hand, the possible mechanism of vasostatin-induced inhibitory effect on VEGF-induced cell proliferation and tube formation in HUVEC was investigated in this study. It was widely known that eNOS and its bioactive product nitric oxide (NO) are well-established proangiogenic molecules. Endothelial-derived NO is crucial for regulation of antiproliferative and antiapoptotic state for EC, and has essential roles in physiological angiogenesis [24–26]. In view of an important role of eNOS in angiogenesis, we evaluated the whether eNOS plays a role on anti-proliferation effect of vasostatin in the present study. As shown in the results, vasostatin decreased eNOS expression in HUVEC dose-dependently, and thereby induced suppression of VEGF-induced increase in cell viability. One eNOS inhibitor, NG-nitro-l-arginine methyl ester (l-NAME), could inhibit endothelial cell migration and proliferation [27]. Thus, we speculated whether vasostatin had a similar mechanism. Disappointingly, there was no significant difference between control and vasostatin-treated groups (Data not shown). Together results indicated that vasostatin might down-regulate eNOS expression, but not suppress eNOS activity to inhibit angiogenesis in endothelial cells. Vasostatin effects on laminin and integrins, which were highly associated with eNOS expression, have previously been demonstrated [28]. We next overexpressed eNOS in HUVEC. It was found that overexpression of eNOS reversed vasostatin-induced inhibitory effects on cell viability and proliferation. Moreover, vasostatin-induced inhibition of tube formation was also reversed by eNOS overexpression. Together these results indicate that the vasostatin-induced inhibitory effect on VEGF-induced cell proliferation and tube formation in HUVEC could be via down-regulation of eNOS expression. Angiogenesis is crucial in tumor growth to acquire adequate blood supply [29], thus, inhibition of angiogenesis could be beneficial for tumor therapy. It is suggested that vasostatin could regulate endothelial cell function and the possibility that vasostatin could be used as an anti-angiogenic agent in cancer deserves future investigations.

## 3. Experimental Section

### 3.1. Cell Isolation and Cultures

Human umbilical vein endothelial cells (HUVEC) were isolated from human cord following the protocol as described [30]. Briefly, collagenase was used to digest HUVEC, then the cells were cultured in 1% gelatin-coated flasks (BD Falcon, Shanghai, China) using Medium 199 (endotoxin-free, Gibco, Grand Island, NY, USA) containing 20% fetal bovine serum (FBS, Hyclone, Logan, UT, USA), 1% bovine retinal-derived growth factor, 90 g/mL heparin, 100 IU/mL penicillin and 100 g/mL streptomycin (Hyclone, Logan, UT, USA). The vasostatin peptide was purchased from Bioshune Bio (Shanghai, China). Briefly, human vasostatin cDNA was cloned into the Pichia Multicopy Expression vectors pPIC9K (Invitrogen, Grand Island, NY, USA). After DNA sequencing, the exogenous genes were transformed into *Pichia* yeast competent cell KM71. The expression of recombinant vasostatin protein was induced and purified with immobilized metal affinity chromatography. The purity of product was over 95%. All experiments were carried out with HUVEC at passage 2–4. For oxygen deprivation, cells were exposed to 95% N_2_ and 5% CO_2_ in a hypoxia gas chamber (Russkin, Bridgend, UK) in full cultural medium for 24 h. Then the following experiments were performed.

### 3.2. Cell Immunofluorescence and MTT Assay

The primary cultured HUVEC were stained with CD31 to identify the purity of HUVEC before experiments. Cells were planted on glasses in six-well plates at 500,000 cells per well and fixed overnight in 4% paraformaldehyde to perform immunofluorescence. The cells were washed with phosphate buffered saline (PBS) for 3 times and blocked in 1% bovine serum albumin (BSA) for 15 min at room temperature. The monoclonal primary antibody rabbit anti-CD31 (1:100, ab76533, abcam, Cambridge, UK) was added on the glasses and incubated overnight at 4 °C. To determine the specificity of immunofluorescence, an isotype control, rabbit IgG (sc2027, Santa Cruz, CA, USA) was used. Following multiple PBS washes, the cells were then incubated with the Alexa Fluor 488-conjugated goat anti-rabbit IgG (1:400, A11008, Invitrogen, Grand Island, NY, USA) for 2 h at room temperature in the dark. After PBS washes, the nuclei of cells were stained by 4′,6-diamidino-2-phenylindole (DAPI). The images were captured by a microscope (DP70, Olympus, Tokyo, Japan).

The cell viability was determined by MTT assay. HUVEC were planted in 96-well plates (15,000 cells per well). Vasostatin was added into the medium in different concentrations (Figure 1) under hypoxic conditions for 24 h. Following treatment, the MTT reagents (3-(4,5-dimethylthiazol-2-yl)-2,5-diphenyl tetrazolium bromide, 0.5 mg/mL) was added to each well for 4 h, then the insoluble purple formazan product was dissolved by dimethyl sulfoxide (DMSO). The absorbance at a wavelength of 490 nm was measured by multiskan (MK3, Thermo, San Jose, CA, USA). For parallel, a heat-inactivated vasostatin peptide had been used for control peptide with similar molecular weight in all experiments.

### 3.3. 5-Ethynyl-2′-deoxyuridine (EdU) and TUNEL Staining

HUVEC were seeded into 24-well plates, and the cells were then incubated with 10 ng/mL concentration of VEGF alone or combined with different concentrations of vasostatin (0.1, 1, 10 mg/mL) for 24 h. Then, the nucleoside analogue EdU (5-ethynyl-2′-deoxyuridine) kit (Invitrogen, Grand Island, NY, USA) was used as the protocol described to determine the proliferation rates in different treated groups. After EdU staining, the images were captured by a microscope (DP70, Olympus, Tokyo, Japan). The proliferation rate of HUVEC was calculated as EdU-positive cells divided by total cells (DAPI-stained).

For TUNEL staining, the cells were cultured on cover glasses in 24-well plates and treated with different concentration of vasostatin (0.1, 1, 10 mg/mL) under hypoxia condition for 24 h. After treatment, the apoptotic cells were stained by TUNEL kit (Roche, Indianapolis, IN, USA) followed its protocol. The apoptotic cells were marked with green fluorescence, and all the cells were stained with DAPI. The ratio of apoptosis was calculated as the apoptotic cells divided by total cells.

### 3.4. In Vitro Morphogenesis and Tube Formation Assay

The tube formation assay was performed as described [31]. Briefly, 4 mg/mL matrigel (BD Biosciences, San Jose, CA, USA) solved in Dulbecco’s PBS (DPBS, Hyclone, Logan, UT, USA) was added into 24-well plates, and the plates were incubated at 37 °C for 30 min to allow gel formation. HUVEC (20,000/well) in Medium 199 containing 1% FCS with VEGF (10 ng/mL) and/or different concentration of vasostatin (0.1, 1, 10 mg/mL) were then plated on the matrigel and incubated overnight, 2-dimensional organization and the network growth area of the cells were photographed using an inverted phase contrast photomicroscope (DP70, Olympus, Tokyo, Japan).

### 3.5. Western Blot Analysis

After different treatments, detailed in the figure legends, the cells were lysed by RIPA lysis buffer (Beyotime, Shanghai, China) according to the manufacturer’s protocol. Western blot was carried out as described with minor modification [32]. The primary antibodies used in this study were cyclin D3 (1:1000, sc182, Santa Cruz, CA, USA), Bcl-2 (1:1000, 2870S, Cell Signaling, Beverly, MA, USA), BAX (1:1000, 5023S, Cell Signaling, Beverly, MA, USA), cleaved caspase8 (1:500, 9496S, Cell Signaling, Beverly, MA, USA), cleaved caspase3 (1:500, 9664S, Cell Signaling, Beverly, MA, USA), β-actin (1:5000, A1978, Sigma Aldrich, St. Louis, MO, USA), eNOS (1:100, sc654, Santa Cruz, CA, USA) respectively. The results of the blot were analyzed by Image J (National Institutes of Health, Bethesda, MD, USA) as optical density (OD).

### 3.6. Transient Transfection of pcDNA3.1-eNOS Plasmid

To overexpress eNOS in primary cultured HUVEC, pcDNA3.1 vector containing the *eNOS* gene (Addgene, Cambridge, MA, USA) was used following the manufacturer’s protocol. Briefly, HUVEC were transfected with plasmids containing the *eNOS* gene by the FUGENE HD transfection kit (Roche, Indianapolis, IN, USA) according to the manufacturer’s instructions. 24 h after transfection, HUVEC were treated with/without 10 ng/mL of VEGF in the presence or absence of 1 mg/mL vasostatin for 24 h. The expression of eNOS protein was examined by western blot to identify the effective of pcDNA3.1-eNOS plasmid as described [33,34]. Then the cell viability, EdU assay and western blot were performed as described above. Tube formation was performed after transient transfection of pcDNA3.1-eNOS plasmid and followed with compound incubation.

### 3.7. Statistical Analysis

All the experiments were repeated at least three times. Data were expressed as the mean ± SD. One-Way ANOVA analysis was performed and a value of *p* < 0.05 was considered significant.

## 4. Conclusions

In this study, it was found that vasostatin could inhibit the cell viability of HUVEC and induce cell mitochondrial apoptosis via Bcl-2-BAX stimulated caspase3 activation under oxygen deprivation condition. Meanwhile, vasostatin also inhibits vascular endothelial growth factor (VEGF)-induced proliferation and tube formation of HUVEC. The possible mechanism of vasostatin-inhibited proliferation of HUVEC could be through eNOS. These findings suggest that vasostatin could regulate endothelial cell function and might be used in anti-angiogenesis treatment.
